# Zoledronate Attenuates Angiogenic Effects of Angiotensin II-Stimulated Endothelial Progenitor Cells via RhoA and MAPK Signaling

**DOI:** 10.1371/journal.pone.0046511

**Published:** 2012-10-11

**Authors:** Jin-Xiu Yang, Bin Chen, Yan-Yun Pan, Jie Han, Fei Chen, Shen-Jiang Hu

**Affiliations:** Institute of Cardiology, The First Affiliated Hospital, College of Medicine, Zhejiang University, Hangzhou, Zhejiang, China; University of Padova, Medical School, Italy

## Abstract

**Background:**

New vessel formation plays a pivotal role in the pathogenesis of neovascular-related diseases. Endothelial progenitor cells (EPCs) were found to contribute to neovascular-related diseases and interference with EPC neovascularization may be a novel target for these diseases. Zoledronate (Zol) was reported to exhibit anti-angiogenic effect. Basing on these evidences, we proposed that Zol may affect EPC function to exert novel anti-angiogenic effect. In this study, we therefore investigated the effects of Zol on multiple aspects of EPC function and explored the underlying mechanisms involved.

**Methodology/Principal Findings:**

EPCs were cultured from bone marrow derived mononuclear cells. The potential effects of Zol on Angiotensin II (Ang II)-stimulated EPC proliferation, migration, adhesion, *in vitro* tube formation were investigated. The results showed that Ang II (1 µM) enhanced EPC migration, adhesion, *in vitro* tube formation but had no effect on cell proliferation. Zol (75 and 100 µM) inhibited proliferation of EPCs and 50 µM geranylgeranyol (GGOH) could reverse the decrease of EPC proliferation. We found for the first time that Zol (50–100 µM) dose dependently attenuated migration, adhesion, and *in vitro* tube formation of EPCs stimulated by Ang II. GGOH could reverse the attenuation of EPC function induced by Zol. However, Zol did not induce EPC apoptosis. In addition, the underlying mechanisms were determined. The results revealed that Zol markedly down-regulated active RhoA stimulated by Ang II and inhibited the phosphorylation of Erk1/2 and JNK. Moreover, RhoA silencing resulted in a notable inhibition of EPC *in vitro* tube formation, suggesting that RhoA suppression played a pivotal role in Zol antiangiogenic effect.

**Conclusions/Significance:**

These findings suggested that Zol attenuated the promotion of EPC function stimulated by Ang II and exhibited novel antiangiogenic effect via RhoA and MAPK signaling. Thus, Zol may be served as a novel therapeutic agent for neovascular-related diseases treatment.

## Introduction

Neovascularization is involved in lots of disorders including diabetic retinopathy [1], cancer [2] and rheumatoid arthritis [3]. Also there is evidence that new vessel formation plays a pivotal role in the pathogenesis of atherosclerosis [4], [5]. Furthermore, the extent of vasa vasorum neovascularization correlates with severity of atherosclerosis [6], [7]. Recent studies have indicated that endothelial progenitor cells (EPCs) contributed to those pathological angiogenesis [8], [9], [10], [11].

EPCs comprise a group of cells that have the capacity to circulate, proliferate and differentiate into mature endothelial cells (ECs) but that have not yet acquired characteristic mature endothelial markers nor formed a lumen [12]. These precursor cells not only participate in angiogenesis, but also contribute to postnatal vasculogenesis [12], [13]. Interference with EPC neovascularization may be a novel target for neovascular-related diseases.

Angiotensin II (Ang II), the main effector of the renin-angiotensin system, has emerged as one of the essential links in the pathophysiology of many neovascular-related diseases such as atherosclerosis and cancer. Ang II was produced increasely within atherosclerotic lesions [14], and was found to induce atherosclerotic plaque vulnerability [15]. Ang II was found to be involved in many types of cancer [16], [17]. Emerging evidence indicated that Ang II increased vessel density and activated angiogenesis [18], thus may aggravate the process of neovascular-related diseases.

Zoledronate (Zol), a third-generation bisphosphonate, has already been approved for clinical use in the treatment of hypercalcemia caused by malignancy [19]. Zol was found to improve atherosclerotic risk index [20] and the prognosis of cancer [21]. Recent studies demonstrated the antiangiogenic effect of Zol [22]. Thus, antiangiogenesis may contribute to Zol protective effects on neovascular-related diseases. It was proposed that Zol exerted antiangiogenic effect through its impact on various angiogenic properties of ECs [23]. Wood et al. reported that Zol could impair EC proliferation, induce EC apoptosis, and modulate EC migration and adhesion, thus exerting antiangiogenic effect [24]. Yamada et al. found that Zol impaired EPC differentiation in a dose-dependent manner, the effect was observed even at low concentrations [22]. At high drug levels, Zol also induced EPC apoptosis [22]. These data suggested that Zol may influence EPC function. However, a thorough analysis of the effect of Zol on multiple aspects of EPC function, especially the molecular mechanisms involved has not been undertaken.

It was found that Zol modulated mevalonate pathway and affected small-G-protein prenylation [25]. RhoA is a very important member of small-G-protein and its most widely studied effector protein is Rho-associated kinases (ROCK) [26]. RhoA/ROCK plays an important role in angiogenesis and may be a significant target for antiangiogenic treatment [26]. RhoA/ROCK has also been indicated as an upstream regulator of mitogen-activated protein kinase (MAPK) family members including extracellular signal-regulated protein kinase (ERK), c-Jun-NH2 kinase (JNK), and p38 MAPK (p38) [27], [28], [29].

Therefore, in this study we investigated the potential effects of Zol on the function of Ang II-stimulated EPCs, and also explored the role of RhoA and MAPK signaling in this process.

## Materials and Methods

### Isolation and cultivation of EPCs

Our study was approved by the institutional animal care committee of Zhejiang University (approval ID: SYXK 2007–2122). Male Sprague-Dawley rats of 6 to 7 weeks old (200 g) were fed with conventional diet.

*In vitro* expansion of rat bone marrow-derived EPCs was performed as recently described [30]. Briefly, EPCs were collected from the femurs of 6 to 7 weeks old male Sprague-Dawley rats (200 g). The mononuclear cells (MNCs) fraction was obtained by density gradient centrifugation. Cells were then suspended in EBM-2 medium (Lonza) supplemented with 10% FBS (Gibico) and plated on 6-well plates (Corning). After 24 h the non-adherent cells were aspirated and transferred to new plates. After another 24 h this procedure was repeated to remove rapidly adherent mature ECs and hematopoietic cells being possibly present in the aspirate. Only the non-adherent cells harvested after 48 h were evaluated further in all experiments. This fraction was cultured in EBM-2 medium supplemented with EGM-2 MV single aliquots containing 10% FBS, vascular endothelial growth factor (VEGF), epidermal growth factor, fibroblast growth factor-2, insulin-like growth factor-1 (IGF-1) and ascorbic acid. Non-adherent cells were removed by washing after 4 d in culture and new media was applied every 3 days.

### EPC fluorescent staining

Fluorescent chemical detection of EPCs was performed on attached MNCs after 14 d in culture. Direct fluorescent staining was used to detect dual binding of 1, 1- dioctadecyl-3, 3, 3, 3-tetramethylindocarbocyanine (DiI)-labeled acetylated low-density lipoprotein (acLDL; Molecular Probe) and fluorescein isothiocyanate (FITC)-conjugated Ulex europaeus agglutinin (UEA)-I (Sigma). The cells were first incubated with acLDL (2.4 µg/ml) at 37°C and later fixed with 2% paraformaldehyde for 10 min. After washing, EPCs were reacted with UEA-I (10 µg/ml) for 1 h. After staining, samples were viewed with fluorescence microscopy (×200). Fluorescence microscopy identified double-positive cells as EPCs.

### Flow cytometry cell analysis

To identify EPCs, the cells were trypsinized, followed by repeated gentle flushing through a pipette tip. Cells (2×10^5^) were incubated for 30 min at 4°C with anti-vascular endothelial growth factor receptor 2 (anti-VEGFR2, Abcam) and phycoerythrin-conjugated monoclonal antibodies against CD31 (R&D), and von Willebrand factor (vWF, Abcam). A FITC-conjugated antimouse antibody (Vector) was added for staining with VEGFR2. Isotype-identical antibodies served as controls. Flow cytometric analyses were performed by using a FACStar flow cytometer (Beckman Coulter).

### Cell proliferation assay

The effect of Zol and Ang II on EPC proliferation was determined by 3-(4, 5-dimethylthiazol -2-yl)-2, 5-diphenyltetraZolium bromide (MTT) assay. Cells were digested with 0.25% trypsin and then cultured in EBM-2 medium containing 10% FBS in 96-well culture plate (200 µl/well). After being cultured for 48 h, the supernatant was discarded by aspiration and serum-free EBM-2 medium was added (200 µl/well). Zol (0, 25, 50, 75, and 100 µM) was added respectively. Geranylgeranyol (GGOH) is metabolized to geranylgeranylpyrophosphate (GGPP) and is used to form geranylgeranylated Rho. It is reported that Zol reduced the synthesis of GGPP and GGOH might restore the reduction of GGPP [31]. Therefore, the group of Zol combined with GGOH was also established. After being incubated for 30 min, EPCs of each well were treated with Ang II (1 µM) and cultured for 48 h. Then they were supplemented with 20 µl MTT (5 g/l, Fluka Co. Product) and incubated for another 4 h. The supernatant was aspirated and the EPCs preparation was shaked with 150 µl dimethyl sulfoxide (DMSO) for 10 min, before the OD value was measured at 490 nm.

### Migration assay

EPC migration was evaluated by using a transwell chamber (Bioscience) with 8-µm pore filters. In brief, isolated cells were detached using 0.25% trypsin, harvested by centrifugation, resuspended in 500 µl EBM-2 medium and counted, then 2×10^4^ EPCs were placed in the upper chamber of a transwell chamber. VEGF in serum-free EBM-2 medium was placed in the lower compartment of the chamber. After 24 h incubation at 37°C in 5% humidified CO_2_, the lower side of the filter was washed with PBS and cells remaining on the upper face were removed with a cotton wool swab. Transwell filters were fixed with 2% paraformaldehyde. For quantification, EPCs were stained with 0.1% crystal violet solution. Cells migrating into the lower chamber were counted in five random microscopic fields (×200).

### EPC adhesion assay

After incubating with Zol, Ang II and GGOH overnight, EPCs were washed with PBS and gently detached with 0.25% trypsin. After centrifugation and resuspension in EBM-2 medium, identical cells were replated onto fibronectin-coated culture dishes and incubated for 30 min at 37°C in 5% humidified CO_2_. Adherent cells were counted by independent blinded investigators in five randomly chosen microscopic fields (×400) [31].

### *In vitro* tube formation assay

Endothelial tube formation was assessed with the use of Matrigel assay (Millipore), according to the manufacturer's instructions. Briefly, ECMatrix™ solution was thawed on ice overnight, mixed with 10×ECMatrix™ diluent and placed in a 96-well plate at 37°C for 1 h to allow the matrix solution to solidify. EPCs were stained with Dil-acLDL (Molecular Probes, 5 µg/ml) for 1 h. Then EPCs (1×10^4^ cells) were harvested and replated on top of the solidified matrix solution with 150 µl EBM-2 medium. Cells were incubated at 37°C for 18 h and fixed with 2% paraformaldehyde. The lengths of enclosed tubes within the network were measured from five random microscopic fields (×200). The experiment was repeated 5 times.

### EPC apoptosis assay

The externalization of phosphatidylserine during apoptosis was evaluated by FITC-conjugated annexin-V and propidium iodide (PI) staining by using an Annexin V-FITC Apoptosis Kit (KeyGEN). After the indicated time, 5×10^5^ cells treated or untreated were harvested, washed twice with PBS, cells were re-suspended in 500 µl binding buffer and 5 µl FITC-labeled annexin-V and 5 µl PI solution were added. Then tubes were kept on ice for 10 min and then subjected on the flow cytometer (Beckman Coulter) which was settled on cells gated on the basis of their forward and side light scatter with any cell debris excluded from analysis. The gated cells were then plotted for FITC-conjugated annexin-V and PI in a 2-way dot plot to assess percentage of apoptotic cells.

### Lentivirus vectors for RhoA small hairpin RNA and cell infection

A third generation self-inactivating lentivirus vector containing a CMV-driven GFP reporter and a U6 promoter upstream of the cloning restriction sites (BamHI and EcoRI) was used. Four coding regions corresponding to targeting rat RhoA starting at positions 153, 81, 354 and 40 in the sequence (GenBank Accession: NM_057132.3) were selected as siRNA target sequences under the guide of siRNA designing software offered by GenePharma Co., Ltd, Shanghai, China. Four shRNA-RhoA lentivirus vectors were respectively constructed by Genepharma. The Lv-NC-shRNA which included the GFP gene was designed with a randomly chosen nonsense sequence to serve as negative control. The most active shRNA against the rat RhoA target DNA sequence, 5′-GATCCGCAGGTAGAGTTGGCTTTATGTTCAAGAGACATAAAGCCAACTCTACCTGCTTTTTTG-3′, which starting at position 153 was selected. Recombinant lentivirus vectors were produced by co-transfecting 293T cells with the lentivirus expression plasmid and packaging plasmids. The virus titers produced were approximately 10^9^ transducing U/ml medium.

When the cells were about 80% confluent in EBM-2 medium, they were detached and subcultured at 1×10^6^ cells per well into six-well plates. After 24 h culture, cells were infected with recombinant lentivirus vectors at a multiplicity of infection (MOI) of 100. Polybrene was added to each well at a concentration of 5 µg/ml.

### RhoA activity assay

RhoA activity was determined by pull-down assay using a Rho activation assay kit (Cytoskeleton) [32]. EPCs were grown to 60% confluence in 100-mm dishes and then placed in serum-free EBM-2 medium for 12 h. Cells were stimulated with 1 µM Angiotensin II (Ang II) for 15 min at 37°C. Zol (50 µM) with or without GGOH (50 µM) were added 48 h before stimulation. Cells were then lyzed with lysis buffer and a protein assay was performed prior to the pull-down assay to equalize total protein concentration in each treated group. Whole cell lysates were incubated with agarose-conjugated rhotekin-RBD at 4°C for 60 min and then washed twice with wash buffer. Agarose beads were boiled in SDS-PAGE sample buffer to release active Rho prior to undergoing precipitation with the Rhotekin GTP-Rho. After precipitation bound Rho proteins were detected by Western blotting using a specific anti-RhoA antibody (Cytoskeleton). Meanwhile, 20 µg total cell lysate per sample was used to detect the total amount of RhoA.

### Western blotting assay

EPCs were grown to 60% confluence in 6 well plate and then placed in serum-free EBM-2 medium for 12 h. Cells were stimulated with 1 µM Ang II for 15 min at 37°C. Zol (50 µM) with or without GGOH (50 µM) were added 48 h before stimulation. The protein contents of the cell lysates were determined using a micro BCA kit (Beyotime). Protein from cell lysates was mixed with 4×loading buffer (Invitrogen) and boiled for 10 minutes, before electrophoresis on 10% sodium dodecyl sulfate-polyacrylamide gels. Following transfer onto polyvinylidene fluoride membranes and blocking, membranes were incubated with antibodies against RhoA, phospho-p38, p38, phospho-JNK, JNK, phospho-ERK1/2 and ERK1/2 (1∶1000, Cell Signaling). Following three washes in TBST, membranes were subsequently incubated with horseradish peroxidase-conjugated goat anti-rabbit IgG antibody (1∶2500, MultiSciences). The signals were detected by enhanced chemiluminescence reagents (Thermo) and exposure to X-ray film. The density of the bands was quantified by using Image J software (National Institutes of Health). Expression of RBD binding RhoA was normalized to total RhoA expression in the same lane and phospho-p38, JNK, ERK1/2 were normalized to total p38, JNK and ERK1/2 respectively. Data from each experiment were presented as percent of control group. In the siRNA experiment, expression of RBD binding RhoA and total RhoA was normalized to β-actin expression in the same lane.

### Statistical analysis

All data are presented as mean ± SD. Differences between group means were assessed by ANOVA for multiple comparisons using SPSS 16.0. Values of P<0.05 were considered significant.

## Results

### Characterization of EPCs

**Figure 1:**
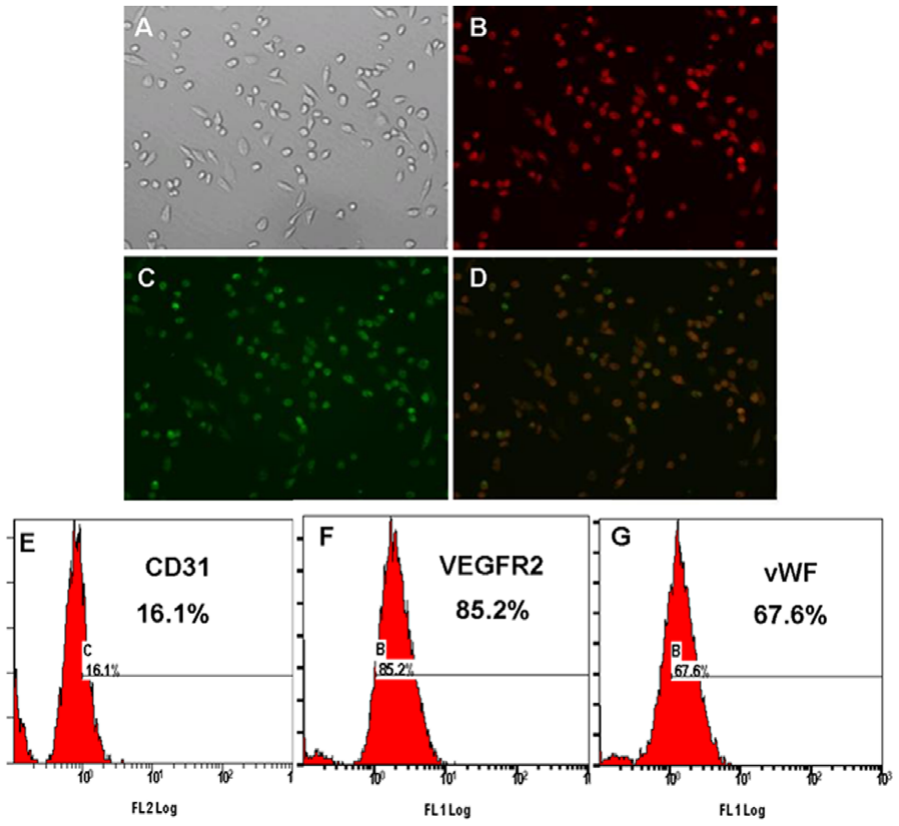
Immunofluorescence identification and immunophenotype of bone marrow derived-EPCs. The attached cells exhibited a spindle shaped, endothelial cells like morphology (A), and adherent cells DiLDL uptake (B: red, exciting wave-length 543 nm) and lectin binding (C: green, exciting wave-length 477 nm) were assessed under a fluorescence microscopy. Double positive cells appearing yellow in the overlay (D) were identified as differentiating EPCs (×200). E to G, Membrane antigen expression on bone marrow-derived EPCs was determined by flow cytometry.

Total MNCs isolated and cultured for 14 d resulted in a spindle-shaped, ECs-like morphology (Figure 1A). EPCs were characterized as adherent cells double positive for DiLDL uptake and lectin binding by using fluorescence microscopy (Figure 1B–D). These cells were characterized further by demonstrating the expression of CD31, VEGFR2 and vWF by flow cytometry (Figure 1E–G).

### Effect of Zol on the proliferation of Zol-pretreated EPCs followed by Ang II stimulation

**Figure 2:**
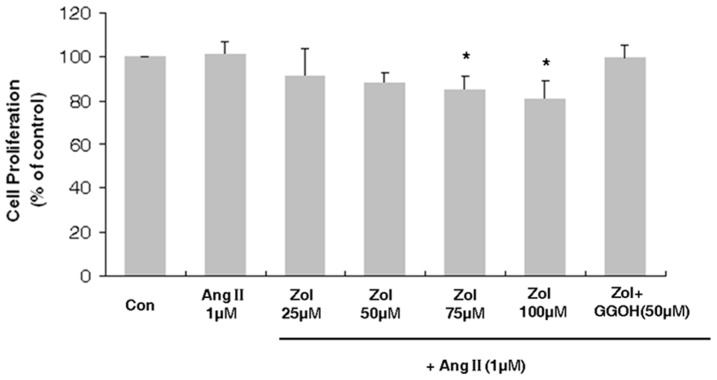
Proliferation of EPCs in response to Ang II and Zol. EPC proliferation was not affected by Ang II, and decreased in presence of Zol and could be partly reversed by GGOH. Data are presented as mean ± SD, n = 5. * P<0.05 vs. Ang II group.

EPC proliferation was measured using the MTT assay (Figure 2). Ang II had no effect on EPC proliferation. Zol reduced EPC proliferative activity, which became apparent at 75 and 100 µM (75 µM Zol+Ang II: 84.83±6.73% of control, 100 µM Zol+Ang II: 80.93±8.36% of control, P<0.05). Although there was no significant statistical difference (P>0.05), GGOH (50 µM) could partly reverse the reduction in EPC proliferation induced by 50 µM Zol (50 µM Zol+Ang II vs. 50 µM Zol+Ang II+50 µM GGOH: 88.37±4.74% of control vs. 99.41±5.94% of control). GGOH (50 µM) could reverse the decreased proliferation of 75 and 100 µM Zol-pretreated EPCs followed by 1 µM Ang II-stimulation (P<0.05, Figure S1).

### Effect of Zol on the migration of Zol-pretreated EPCs followed by Ang II stimulation

**Figure 3:**
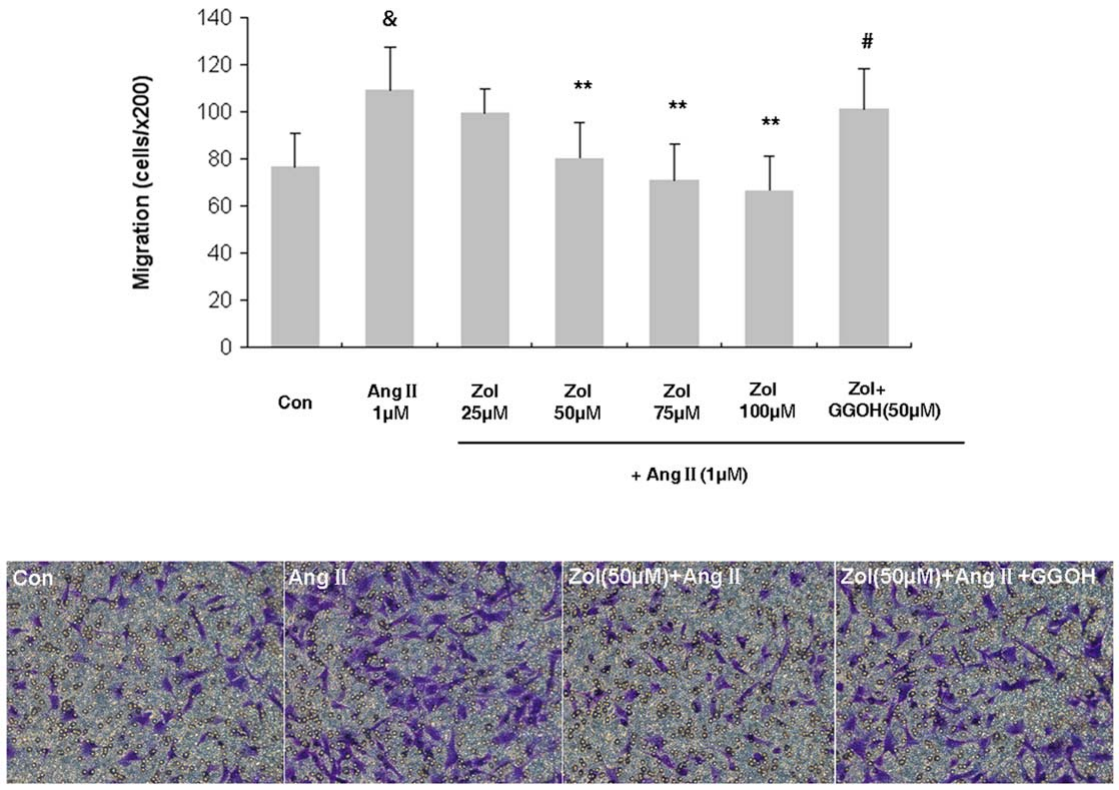
Migration of EPCs cocultured with Ang II and Zol. Migratory effect of EPCs was improved by Ang II, while impaired by Zol dose dependently and this effect could be reversed by GGOH. Data are presented as mean ± SD, n = 5. &P<0.01 vs. control group; ** P<0.01 vs. Ang II group; # P<0.05 vs. 50 µM Zol+Ang II group.

EPC migration was analyzed by the transwell chamber assay (Figure 3). The result showed that Ang II promoted EPC migration (control vs. Ang II: 76.8±14.3 vs. 109.2±18.3, P<0.05). Zol dose dependently decreased EPC migration, which became apparent at 50 µM (Ang II vs. 50 µM Zol+Ang II: 109.2±18.3 vs. 80.2±15.1, cells per high-powered field, P<0.01), with a peak at 100 µM (Ang II vs. 100 µM Zol+Ang II: 109.2±18.3 vs. 66.4±14.9, P<0.01). GGOH (50 µM) could reverse the decrease of EPC migration capacity induced by 50 µM Zol (50 µM Zol+Ang II vs. 50 µM Zol+Ang II+50 µM GGOH: 80.2±15.1 vs. 101.2±17.2, P<0.05).

### Effect of Zol on the adhesion of Zol-pretreated EPCs followed by Ang II stimulation

**Figure 4:**
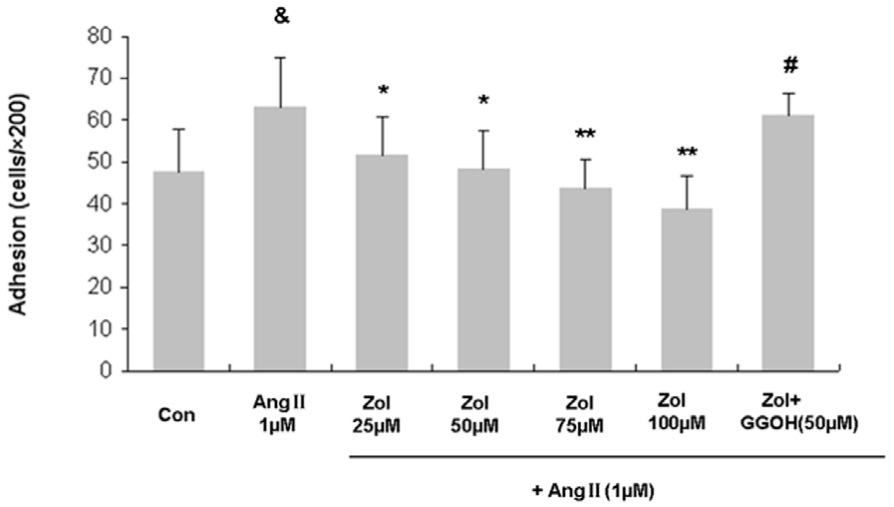
Adhesion capacity of EPCs treated with Ang II and Zol. Ang II increased EPC adhesion capacity, while Zol dose dependently decreased cell adhesion and GGOH showed reversal effect. Data are presented as mean ± SD, n = 5. &P<0.05 vs. control group; * P<0.05 vs. Ang II group; ** P<0.01 vs. Ang II group; # P<0.05 vs. 50 µM Zol+Ang II group.

To study the possibility that Zol alter adhesion of EPCs, cells were incubated with Zol (at a serial of concentration: 0, 25, 50, 75, and 100 µM) and Ang II (1 µM) for 48 h. After replating on fibronectin-coated dishes, EPCs preexposed to Ang II exhibited a significant increase in the number of adhesive cells (control vs. Ang II: 47.6±10.1 vs. 63.0±12.0, P<0.05). EPC adhesion ability was attenuated by Zol in a dose dependent manner, which became apparent at 25 and 50 µM (Ang II vs. 25 µM Zol+Ang II: 63.0±12.0 vs. 51.84±9.4; Ang II vs. 50 µM Zol+Ang II: 63.0±12.0 vs. 48.2±9.1, P<0.05), with a peak at 100 µM (Ang II vs. 100 µM Zol+Ang II: 63.0±12.0 vs. 38.8±7.9, P<0.01) (Figure 4). GGOH (50 µM) could reverse the attenuation of EPC adhesion ability induced by 50 µM Zol (50 µM Zol+Ang II vs. 50 µM Zol+Ang II+50 µM GGOH: 48.2±9.1 vs. 61.0±5.4, P<0.05) (Figure 4).

### Effect of Zol on the *in vitro* tube formation of Zol-pretreated EPCs followed by Ang II stimulation

**Figure 5:**
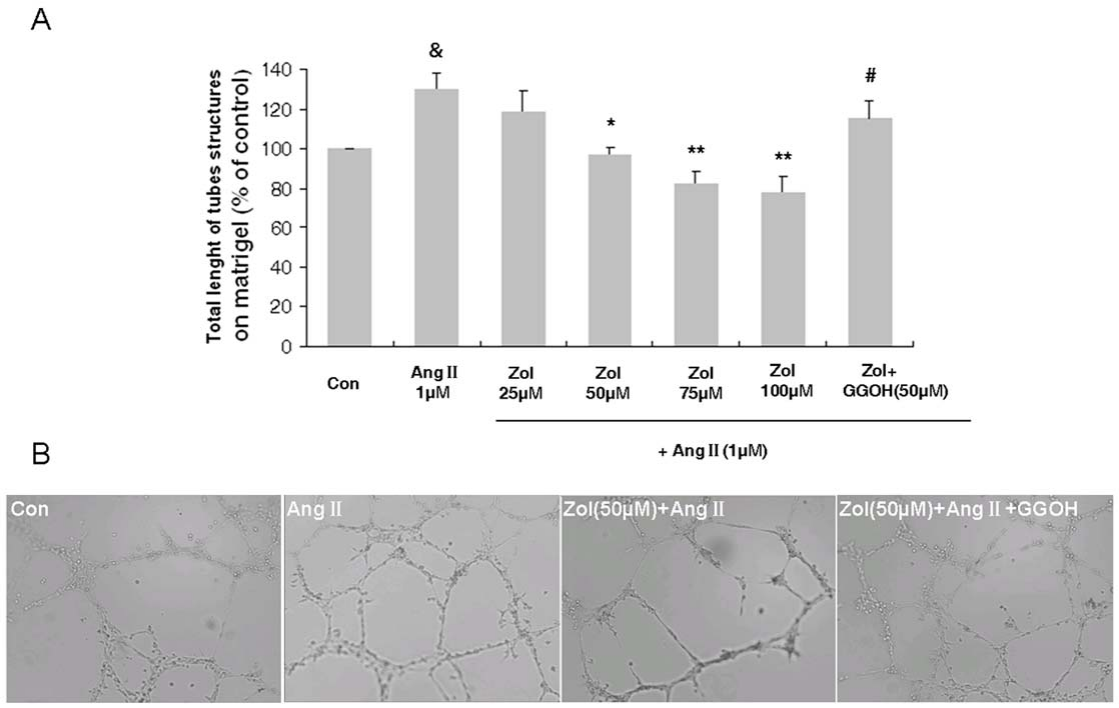
Effect of Ang II and Zol on EPC *in vitro* tube formation ability. Ang II increased EPC *in vitro* tube formation ability, while Zol dose dependently decreased cell *in vitro* tube formation and GGOH showed reversal effect. A: Quantitative analysis of total length of the tubes formed on Matrigel for each experimental group. B: Light micrographs showing typical tubules (200×). Results are the mean ± SD, n = 5. &P<0.01 vs. control group; * P<0.05 vs. Ang II group; ** P<0.01 vs. Ang II group; # P<0.05 vs. 50 µM Zol+Ang II group.

EPCs were incubated overnight in starvation medium and then stimulated for 48 h with Zol and Ang II in basal medium. EPCs were stained with Dil-acLDL for 1 h before seeding on matrigel for 18 h. Ang II profoundly enhanced EPC *in vitro* tube formation (129.86±8.42% of control, P<0.01). Zol decreased EPC *in vitro* tube formation ability in a concentration-dependent manner, which became apparent at 50 µM (Ang II vs. 50 µM Zol+Ang II: 129.86±8.42% of control vs. 97.08±3.48% of control, P<0.05), with a peak at 100 µM (Ang II vs. 100 µM Zol+Ang II: 129.86±8.42% of control vs. 77.78±8.27% of control, P<0.01) (Figure 5). GGOH (50 µM) could reverse the inhibition of EPC *in vitro* tube formation ability by 50 µM Zol (50 µM Zol+Ang II vs. 50 µM Zol+Ang II+50 µM GGOH: 97.08±3.48% of control vs. 115.47±8.79% of control, P<0.05) (Figure 5).

### Effect of Zol on EPC apoptosis

We used the Annexin V-FITC Apoptosis Kit to investigate the effect of Zol on EPC apoptosis. The result showed that EPC apoptosis was not affected by Zol (Figure S2).

### Effect of Zol on RhoA activation of Zol-pretreated EPCs followed by Ang II stimulation

**Figure 6:**
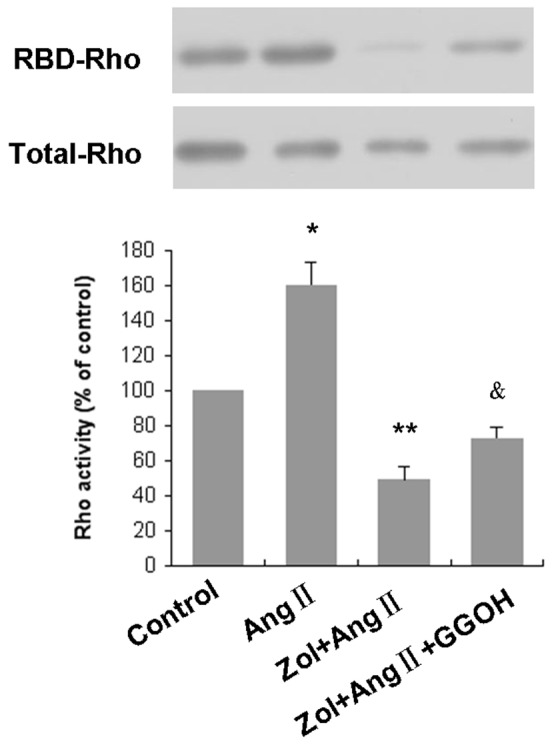
Effect of Zol on EPC RhoA activation. Serum-starved EPCs in the absence or presence of 50 µM Zol were combined with or without 50 µM GGOH before incubation with 1 µM Ang II for 15 min. RBD-RhoA proteins were detected by Western blot using monoclonal antibody against RhoA (upper panel). Western blotting of the total amount of RhoA in cell lysates (lower panel) was also performed in the same lysates. The results are representative of three independent experiments. * P<0.01 vs. control group; ** P<0.01 vs. Ang II group; &P<0.01 vs. Zol+Ang II group.

We used a pull-down assay with the fusion protein GST-RBD, which specifically recognizes Rho-GTP, the active form of Rho to investigate the effect of Zol on RhoA activation of Zol-pretreated EPCs followed by Ang II stimulation. Rho-GTP increased in EPCs treated with Ang II for 15 min. The active form of RhoA (GTP-bound) was elevated to 160.57±12.98% of control after the addition of Ang II (Figure 6). Pretreatment with 50 µM Zol markedly reduced RhoA activation to 49.19±7.79% of control (P<0.01). In the presence of 50 µM GGOH, the inhibitory effect of Zol against the activation of RhoA by Ang II was markedly reversed to 72.94±6.19% of control, but was still significantly lower than the Ang II group (Figure 6). These results suggest that RhoA activation is partially attenuated by Zol via the inhibition of geranylgeranylation.

### RhoA suppression played a pivotal role in Zol antiangiogenic effect

**Figure 7:**
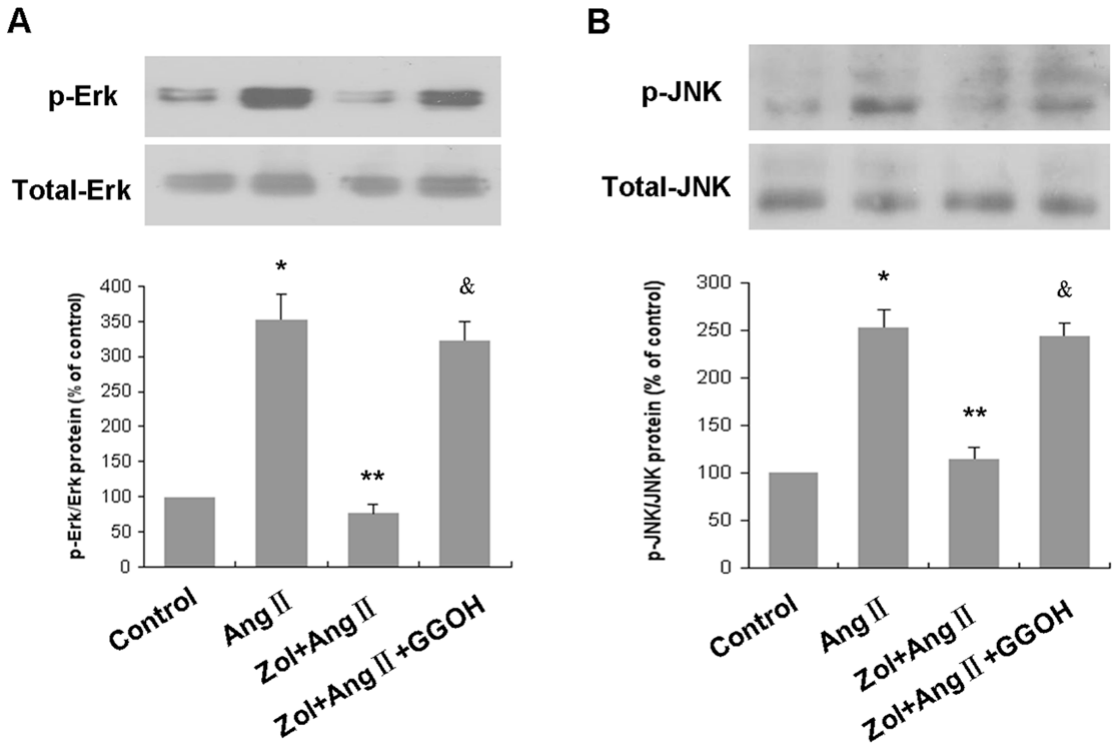
Role of RhoA suppression in Zol antiangiogenic effect. shRNA was used to silence RhoA gene expression. RhoA expression and EPC *in vitro* tube formation were investigated. A–B, RBD-RhoA proteins (upper panel) and the total amount of RhoA (middle panel) were detected by Western blot using monoclonal antibody against RhoA. β-actin was the endogenous control for each sample (lower panel). Values shown are representative of three independent experiments. Control shRNA group: * P<0.01 vs. control group; &P<0.01 vs. Ang II group; RhoA shRNA group: # P<0.01 vs. control group; 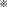  P<0.01 vs. Ang II group; ** P<0.01 for Control shRNA vs. RhoA shRNA group. C–D, Light and fluorescence micrographs showing typical tubules. Quantitative analysis of total length of the tubes formed on Matrigel for each experimental group was performed. The results are representative of three independent experiments. Control shRNA group: ** P<0.01 vs. control group; &P<0.01 vs. Ang II group; RhoA shRNA group: # P<0.05 vs. Ang II group; * P<0.05 for Control shRNA vs. RhoA shRNA group.

To explore the role of RhoA signaling in the antiangiogenic effect of Zol, we used shRNA to silence RhoA gene expression and investigated its effect on EPC *in vitro* tube formation. The data showed that the expression of both total and active form of RhoA was decreased markedly in RhoA shRNA interference group compared with control shRNA interference group (p<0.01, Figure 7). Then we investigated the role of RhoA gene silence in the effect of Zol on EPC angiogenic property. EPCs were stained with Dil-acLDL (5 µg/ml) for 1 h before the measurement of cell *in vitro* tube formation. The results showed that EPC tube formation was promoted by Ang II stimulation while attenuated by Zol in control shRNA interference group. Intriguingly, RhoA silencing resulted in a notable inhibition of EPC *in vitro* tube formation (p<0.05, Figure 7). These results suggested that RhoA suppression played a pivotal role in Zol antiangiogenic effect.

### Zol inhibited JNK and ERK phosphorylation

**Figure 8:**
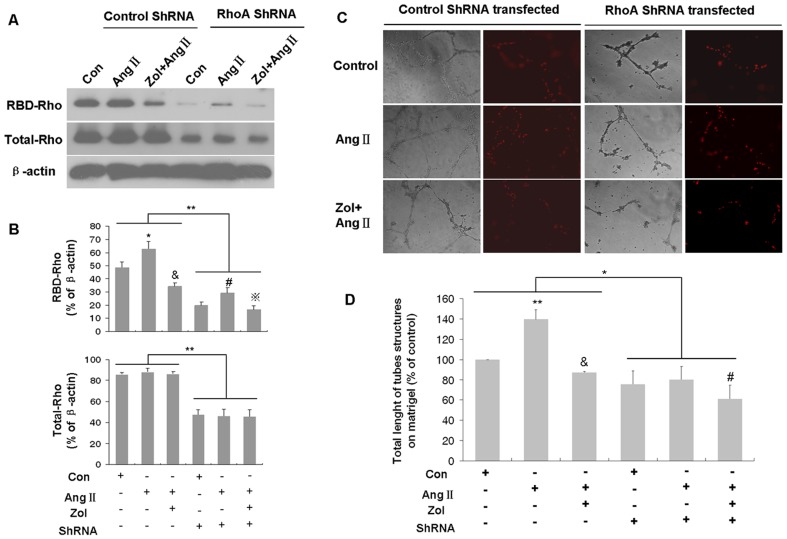
Involvement of ERK (A) and JNK (B) signaling in the effects of Zol on EPCs. Serum-starved EPCs in the absence or presence of 50 µM Zol were combined with or without 50 µM GGOH before incubation with 1 µM Ang II for 15 min. Phosphorylated Erk, total Erk, phosphorylated JNK, and total JNK proteins were detected by Western blot. Values shown are representative of three independent experiments. * P<0.01 vs. control group; ** P<0.01 vs. Ang II group; &P<0.01 vs. Zol+Ang II group.

To investigate the molecular mechanisms further, MAPK pathway was detected in each group using western blot analysis. Erk1/2 phosphorylation was increased to 352.33±36.19% of control and JNK phosphorylation was promoted to 252.99±18.39% of control after administration of 1 µM Ang II. The phosphorylation of Erk1/2 and JNK was markedly reduced in EPCs that pretreated with 50 µM Zol (P<0.01). In the presence of 50 µM GGOH, the inhibitory effects of Zol against the phosphorylation of Erk1/2 and JNK were reversed significantly (Figure 8). Neither total Erk 1/2 nor JNK was changed. There was no obvious influence of Zol on p38 phosphorylation (data not shown). These results suggest the involvement of Erk1/2 and JNK signal pathway in the role of Zol effects on EPCs.

## Discussion

The regulation of neovascularization is an important therapeutic target in neovascular-related diseases. There is considerable data implicating a role for Ang II in the atherogenic process [33]. Vasa vasorum neovascularization plays a pivotal role in the pathogenesis of atherosclerosis [6], [7]. Ang II was also found to be involved in many types of cancer [16], [17]. EPCs participate in pathological angiogenesis which is implicated in the pathogenesis of neovascular-related diseases such as atherosclerosis [3] and cancer [34]. So it is probable that Ang II may contribute to the aggravation of neovascular-related diseases through affecting EPC angiogenetic-related function.

In this study, we found that EPC migration, adhesion capacity, and *in vitro* tube formation ability were promoted by 1 µM Ang II, although EPC proliferation was not affected by Ang II. These findings suggested that Ang II participated in the process of neovascular-related diseases by affecting EPC function, and interference with these function may represent a promising therapeutic strategy in the treatment of neovascular-related diseases.

Zoledronate (Zol), a third-generation bisphosphonate, was seen to improve atherosclerotic risk index [20] and the prognosis of cancer [21]. Zol may have potential therapeutic effects on the treatment of atherosclerosis and tumor. Recent studies have demonstrated anti-angiogenic properties of Zol [22], which may contribute to its protective effects on the treatment of atherosclerosis and tumor. However, the precise mechanisms of anti-angiogenic properties of Zol have not yet been fully clarified.

In present study, we investigated the effects of Zol on function of EPCs stimulated by Ang II. We have demonstrated for the first time that Zol was able to significantly attenuate EPC function, thus exerting anti-angiogenic effect. We found that Zol decreased EPC proliferative activity, and the effect could be reversed by GGOH. In cells, GGOH is metabolized to GGPP which is reduced by Zol [35]. Zol also reduced the promotion of EPC migration ability and adhesion capacity induced by 1 µM Ang II, while GGOH showed reversal effect. The observation that Zol affected EPC proliferation, migration, and adhesion prompted us to investigate the possibility that Zol could also inhibit angiogenic ability. *In vitro* tube formation assay was performed to investigate the effect of Zol on EPC angiogenic ability. The results showed that EPC *in vitro* tube formation was profoundly down-regulated by Zol in a concentration-dependent manner, which became apparent at 50 µM, with a peak at 100 µM. The effect on EPCs was reversed by incubating with 50 µM GGOH. The effect of Zol (25, 50, 75, and 100 µM) on EPC apoptosis was also investigated. The data showed that Zol did not affect EPC apoptosis even at 100 µM, confirming that the decrease in proliferation, migration, adhesion, and *in vitro* tube formation of EPCs treated with Zol was not due to apoptosis of the cells.

Zol is one kind of nitrogen containing bisphosphonates, which inhibits the mevalonate pathway [36], [37]. This pathway is significant for the production of small G proteins, such as Rho, Ras and Rac proteins, which are essential for intracellular structure and function [38], [39]. Substantial proof has accumulated that the Rho proteins play an important role in angiogenesis [40], [41]. It is imperative that we further explore this signaling pathway, as it may be served as an excellent therapeutic target for diseases of aberrant angiogenesis. RhoA (the predominant isoform of Rho: A, B, C) acts as a molecular switch cycling between an inactive guanosine diphosphate (GDP)-bound and an active guanosine triphosphate (GTP)-bound form [42], [43]. RhoA and its effector protein ROCK regulate downstream effectors of MAPK family members including ERK, JNK, and p38 [27], [28], [29]. Ang II was also found to activate RhoA/ROCK signaling [28]. In this study, we further explored the role of RhoA and MAPK signaling in novel antiangiogenic effect of Zol on EPCs stimulated by Ang II.

The active form of Rho (GTP-bound) was elevated after the addition of Ang II. Pretreatment with Zol markedly reduced RhoA activation. In the presence of GGOH, the inhibitory effect of Zol against the activation of RhoA by Ang II was markedly reversed. These results suggested that RhoA activation was attenuated by Zol via the inhibition of geranylgeranylation. Our data also showed that the phosphorylation of Erk1/2 and JNK was increased remarkably compared to control group after administration of Ang II. Zol reduced the phosphorylation of Erk1/2 and JNK of EPCs. GGOH significantly reversed the inhibitive effects of Zol against the phosphorylation of Erk1/2 and JNK. These results suggest the involvement of Erk1/2 and JNK signal pathway in the role of Zol antiangiogenic property. Several studies have shown that MAPK pathway was involved in angiogenesis [44]. Ang II was found to activate MAPK and through this pathway elicited many cellular responses [45]. RhoA/ROCK has also been indicated as an upstream regulator of MAPK family members [27], [28], [29]. Our data suggest that the novel antiangiogenic effect of Zol might be through impairing EPC function via RhoA and MAPK signaling. The direct relationship between these findings was further confirmed by the use of shRNA to silence RhoA gene expression. The data showed that the expression of both total and active form of RhoA was decreased markedly in RhoA shRNA interference group compared with control shRNA interference group. Meanwhile, RhoA silencing resulted in a notable inhibition of EPC *in vitro* tube formation. These results suggested that RhoA suppression played a pivotal role in Zol antiangiogenic effect.

It is worthy to note that the cells cultured in this study were not colony forming unit-Hill (CFU-Hill), although the culture method was similar. CFU-Hill cells were first defined by Hill et al. in 2003 [46]. Human peripheral blood MNCs were resuspended in Medium 199 growth medium and plated on plates coated with human fibronectin. Non-adherent cells were collected 48 hours later and replated onto new fibronectin-coated plates. Seven days later, the numbers of EPCs colony-forming units were counted. The cells cultured in our study have a different origin from that of CFU-Hill cells. In addition, the culture process of the cells was not the same.

In the study, we cultured EPCs with MNCs derived from healthy rats to investigate the antiangiogenic effects of Zol on EPCs in the presence of Ang II. However, if we wanted to study the antiangiogenic effects of Zol on EPCs in atherosclerosis, we should use MNCs derived from atherosclerotic animals. As well, we should test *in vivo* whether Zol could attenuate EPC-induced neovascularization for vasa vasorum of atherosclerotic plaque.

In conclusion, our data showed that Ang II enhanced EPC migration, adhesion, and *in vitro* tube formation. Zol could reverse these effects and reduce cell proliferation, thus exhibiting novel antiangiogenic effect. RhoA and MAPK signaling may be involved in these processes.

## Supporting Information

Figure S1 **Effect of GGOH on the proliferation of Zol-pretreated EPCs followed by Ang II-stimulation.** EPC proliferation was decreased in presence of Zol (75 and 100 µM) and 1 µM Ang II, and could be reversed by 50 µM GGOH. Data are presented as mean ± SD, n = 5. * P<0.05 vs. Ang II group; &P<0.05 vs. 75 µM Zol+Ang II group; # P<0.05 vs. 100 µM Zol+Ang II group.
(TIF)

Figure S2 **Effect of Zol on EPC apoptosis.** (A) Representative dot-plots of apoptotic cells cocultured with different concentrations of Zol. (B) Zol did not affect EPC apoptosis. Data are presented as mean ± SD, n = 3, P>0.05.
(TIF)
