# Supplementary figures and images for: Zoledronate Attenuates Angiogenic Effects of Angiotensin II-Stimulated Endothelial Progenitor Cells via RhoA and MAPK Signaling
Source: PLoS One. 2012 Oct 11;7(10):e46511. doi: 10.1371/journal.pone.0046511 (PMC3469623; doi:10.1371/journal.pone.0046511)

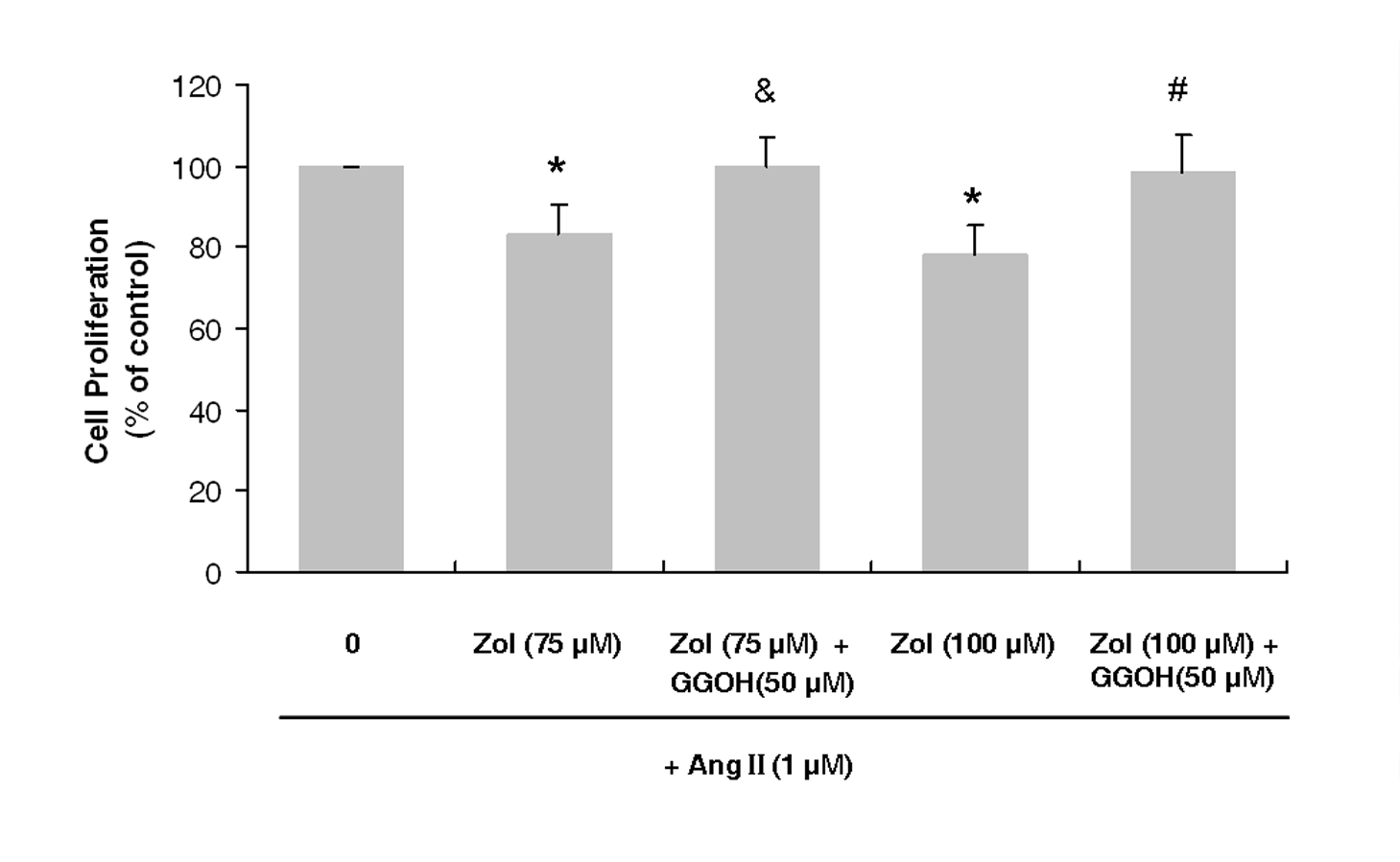

Supplement: Figure S1 — Effect of GGOH on the proliferation of Zol-pretreated EPCs followed by Ang II-stimulation. EPC proliferation was decreased in presence of Zol (75 and 100 µM) and 1 µM Ang II, and could be reversed by 50 µM GGOH. Data are presented as mean ± SD, n = 5. * P<0.05 vs. Ang II group; &P<0.05 vs. 75 µM Zol+Ang II group; # P<0.05 vs. 100 µM Zol+Ang II group. (TIF) [file pone.0046511.s001.tif]

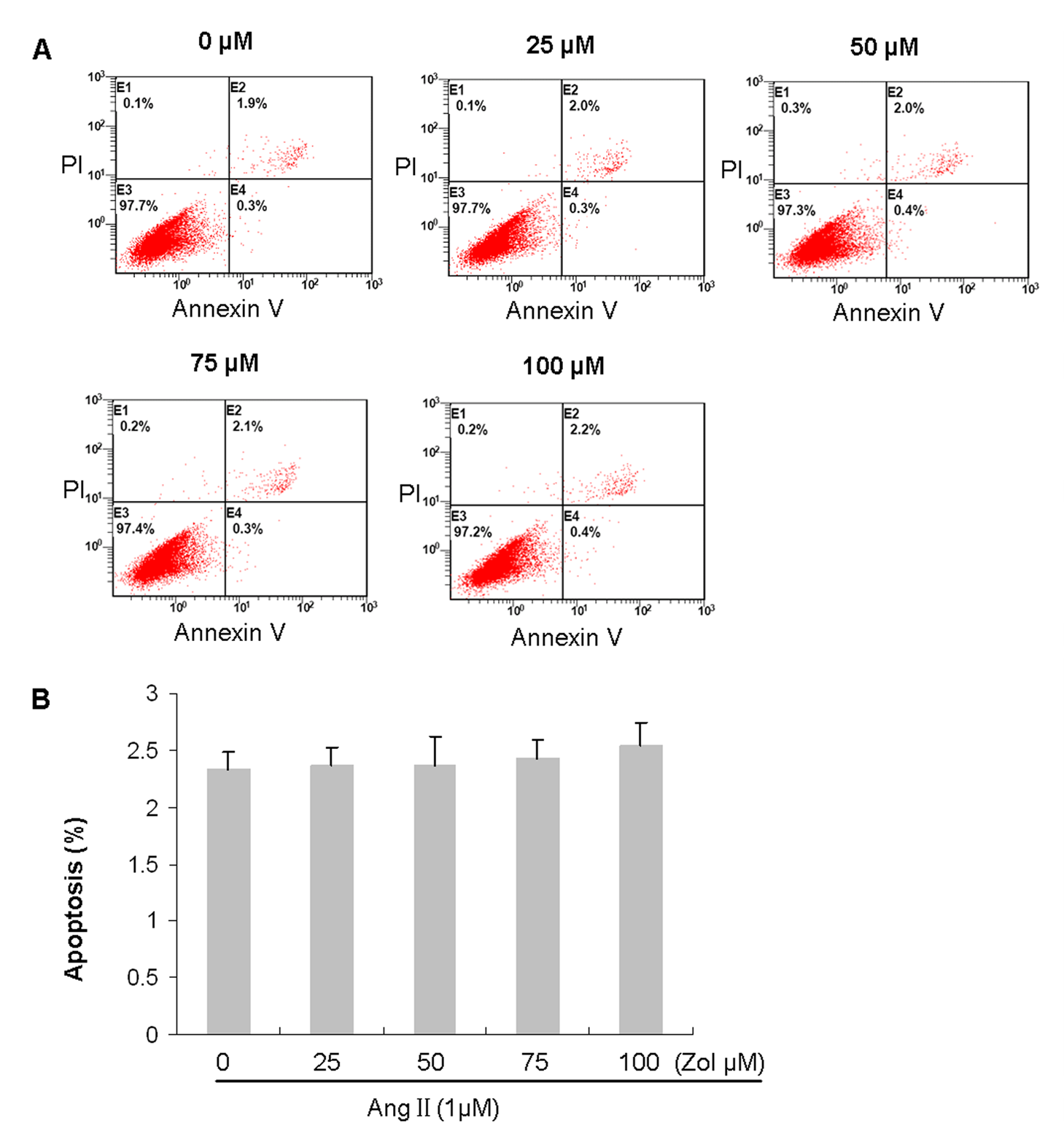

Supplement: Figure S2 — Effect of Zol on EPC apoptosis. (A) Representative dot-plots of apoptotic cells cocultured with different concentrations of Zol. (B) Zol did not affect EPC apoptosis. Data are presented as mean ± SD, n = 3, P>0.05. (TIF) [file pone.0046511.s002.tif]
